# Itraconazole induces apoptosis and cell cycle arrest via inhibiting Hedgehog signaling in gastric cancer cells

**DOI:** 10.1186/s13046-017-0526-0

**Published:** 2017-04-11

**Authors:** Qiang Hu, Yi-Chao Hou, Jiao Huang, Jing-Yuan Fang, Hua Xiong

**Affiliations:** Division of Gastroenterology and Hepatology; Key Laboratory of Gastroenterology and Hepatology, Ministry of Health; State Key Laboratory for Oncogenes and Related Genes; Renji Hospital, School of Medicine, Shanghai Jiao Tong University; Shanghai Institute of Digestive Disease, 145 Middle Shandong Road, Shanghai, 200001 China

**Keywords:** Itraconazole, Gastric cancer, Hedgehog signaling, Gli1

## Abstract

**Background:**

Itraconazole has been proved therapeutically effective against a variety of human cancers. This study assessed the effect of itraconazole on the Hedgehog (Hh) pathway and proliferation of human gastric cancer cells.

**Methods:**

CCK-8 assay and colony formation assay were used to assess the effects of itraconazole on proliferation of gastric cancer cells. The expression of Hh signaling components in gastric cancer cells treated with itraconazole was evaluated by reverse-transcription polymerase chain reaction, immunoblotting and dual luciferase assay. Tumor xenograft models were used to assess the inhibitory effect of itraconazole on the proliferation of gastric cancer cells in vivo.

**Results:**

Itraconazole could remarkably inhibit the proliferation of gastric cancer cells. When in combination with 5-FU, itraconazole significantly reduced the proliferation rate of cancer cells. Furthermore, itraconazole could regulate the G_1_-S transition and induce apoptosis of gastric cancer cells. Hh signaling was abnormally activated in human gastric cancer samples. In vitro, studies showed that the expression of glioma-associated zinc finger transcription factor 1 (Gli1) was decreased at both transcriptional and translational levels after treatment with itraconazole. Dual luciferase assay also indicated that itraconazole could inhibit the transcription of Gli1. In vivo studies demonstrated that monotherapy with itraconazole by oral administration could inhibit the growth of xenografts, and that itraconazole could significantly enhance the antitumor efficacy of the chemotherapeutic agent 5-FU.

**Conclusions:**

Hh signaling is activated in gastric tumor and itraconazole can inhibit the growth of gastric cancer cells by inhibiting Gli1 expression.

## Background

Gastric cancer (GC) has attracted much attention for its high morbidity and mortality worldwide; in the rank of cancer-related death, it lies in the fourth in males and fifth in females [1]. A 5-year survival rate after surgical tumor resection is less than 30% and the response rate to chemotherapy is less than 40% in unresectable and recurrent cases. The mean survival time is approximately 9 months [2]. Until now, the molecular mechanisms responsible for the transformation of gastric cancer are poorly characterized. Thus, it is urgent to find new treatment targets and effective drugs.

The Hedgehog (Hh) signal pathway was firstly identified in Drosophila [3]. Studies suggest that activated Hh signaling is pivotal to the regulation of cell proliferation, cell differentiation and tissue generation during embryogenesis. However, in adult, Hh signaling keeps silent and is highly associated with carcinogenesis once abnormally activated. For example, Hh activation has been reported in basal cell carcinoma, medulloblastoma, non-small cell lung cancer, breast cancer and gastric cancer. A study from Greece demonstrated Hh signaling pathway is activated in non-small cell lung cancer and correlated with histology and prognosis of the tumors [4]. Inhibition of Hh signaling or GLI2 knockdown in human osteosarcoma cell lines reduces cell proliferation and induces apoptosis [5, 6]. Animal experiments also demonstrated that overactivation of Hh pathway due to Ptch1 mutation could induce medulloblastoma and basal cell carcinoma in mouse model [7, 8].

As a traditional antifungal drug, itraconazole is a Food and Drug Administration Agency-approved agent to treat systemic fungal infections, especially in immunocompromised and cancer patients [9, 10]. Intriguingly, in a process of screening of old drugs for novel biological functions, itraconazole, unlike other azole antifungals, has been found to have a function to inhibit endothelial cell proliferation and angiogenesis [11, 12]. Increasing studies have shown that itraconazole may be an effective adjuvant to current treatment in selected cancers [13–18]. Antonarakis et al. demonstrated that itraconazole at high-dose (600 mg/day) has modest antitumor activity in men with metastatic castration-resistant prostate cancer that is not mediated by testosterone suppression [13]. Another phase II clinical trial showed that itraconazole can reduce basal cell carcinoma tumor size after 1 month of treatment [14]. Using primary xenograft models of human non-small cell lung cancer, Blake et al. found that oral administration of itraconazole alone could inhibit tumor growth associated with induction of hypoxia-inducible factor 1 alpha expression and marked inhibition of tumor vascularity [19]. In addition, itraconazole has been included in coordinated undermining of survival paths with nine repurposed drugs (CUSP9) by the International Initiative, which is a conceptually new treatment approach for relapsed glioblastoma [20, 21]. Notably, it has also discovered recently that itraconazole can potently inhibit Hh signaling, of which the mechanism is clearly different from its inhibitory effect on fungal ergosterol synthesis [22].

In this study, we explored the anti-tumor effects of itraconazole on gastric cancer and its regulatory effect on Hh signaling pathway. Firstly, we employed several in vitro assays using MKN45 and AGS cells to evaluate the effect of itraconazole on gastric cancer cell functions, including proliferative capacity, cell cycle and cell apoptosis. The effect of itraconazole on Hh signaling in gastric cancer was further evaluated. The results demonstrated that itraconazole could inhibit proliferation of gastric cancer cells by inhibiting Gli1 expression. These results provide a theoretical foundation for application of itraconazole to the anticancer therapy.

## Methods

### Cell culture and reagents

Two gastric cancer cell lines, MKN45 (KCLB 80103) and AGS (ATCC CRL 1739), were maintained in RPMI-1640 medium (Gibco) supplemented with 10% fetal bovine serum (ScienCell) and 1% penicillin-streptomycin sulfate (Gibco). All cultures were maintained in a 37^。^C incubator supplemented with 5% CO_2_. Itraconazole (Sigma) was dissolved in dimethyl sulfoxide (DMSO) for in vitro experiments. Itraconazole oral solution (H20080401, Xi’an Janssen Pharmaceutica Ltd) and 5-FU (APP Pharmaceuticals) for in vivo experiments were obtained from the pharmacy of Renji Hospital.

### Proliferation assays

MKN45 and AGS cells (3× 10^3^ cells/well) were seeded onto 96-well plates. After cells adhered, itraconazole or equal volume of vehicle DMSO was added to the cells, and the cells were cultured for 24, 48 or 72 h. Ten μl CCK-8 solution from Cell Counting Kit 8 (CCK-8, Dojindo, Japan) was added to each well and the plate was incubated at 37 °C for an additional 1 h. The cell viability was calculated as OD value at a wavelength of 450 nm according to the manufacturer’s instructions.

### Colony formation assays

For colony formation assay, MKN45 and AGS cells were plated onto a 6-well plate at a density of 800 or 500 per well separately. Different concentrations of itraconazole solution and DMSO were added to the medium after cells adhered. The cells were continuously cultured for approximately 2 weeks until evident colony formation was observed. Colonies were fixed with 75% methanol and stained with 1% crystal violet (BIO BASIC INC). Only those cell clusters containing more than 50 cells under a microscope were considered as colonies. The assay was performed in triplicate.

### Cell cycle and cell apoptosis analysis

MKN45 and AGS cells were seeded onto a 6-well plate and were cultured for 72 h under the conditions as indicated. For cell cycle analysis, the cells were harvested by trypsinization and fixed in 70% ice-cold ethanol at −20 °C overnight. Cells were then centrifuged, washed with PBS and suspended in 100 μg/ml RNase A (Thermo Scientific, EN0531) for 30 min at 37 °C. Cellular DNA was stained with 50 μg/ml propidium iodide (PI) (Sigma, P4170) for 10 min at 4 °C in the dark before analysis by flow cytometry. Cell apoptosis was determined using FITC Annexin V Apoptosis Detection Kit II (BD Biosciences). Cells were harvested and suspended in Annexin V Binding Buffer. Then FITC Annexin V and PI solutions were added in sequence according to the manufacturer’s instructions. After incubation, the stained cells were analyzed by flow cytometry. A total of 50,000 events were analyzed for each sample with a FACS Calibur (Becton Dickinson) for determination of cell cycle profiles and apoptotic cells.

### Real-time reverse transcription-polymerase chain reaction (real-time RT-PCR)

Total RNA was isolated from MKN45 and AGS cells using Trizol (RNAiso plus) (Takara, 9109). RNA was reverse-transcribed into cDNA using the Reverse Transcription Kit (Takara, RR03TA) as directed by the manufacturer. Quantitative real-time PCR was performed using SYBR® Premix Ex Taq™ II (Takara, DRR820A) in an ABI StepOnePlus system (Applied Biosystems Inc.). Amplification conditions were 35 cycles at 3 different temperatures, 95 °C for 30 s, 55 °C for 5 s, and 72 °C for 5 s; after amplification, melting curves were analyzed to confirm the specificity of amplicons. The primer sequences were as follows: Shh, 5′-CAGTGGACATCACCACGTCT-3′ and 5′-CCGAGTTCTCTGCTTTCACC-3′; Ptch1, 5′-GGCAGCGGTAGTAGTGGTGT-3′ and 5′-CGGGTATTGTCGTGTGTGTC-3′; Ptch2, 5′-GTGTGGTGGGAGGCTATCTG-3′ and 5′-GGGTAGTGGCAGCATTGAAG-3′; Smo, 5′-CTATTCACTCCCGCACCAAC-3′ and 5′-CAGTCAGCCCACAGGTTCTC -3′; Gli1, 5′-GAAGTCATACTCACGCCTCGAA-3′ and 5′-CAGCCAGGGAGCTTACATACAT-3′; GAPDH, 5′-GCATTGCCCTCAACGACCAC-3′ and 5′-CCACCACCCTGTTGCTGTAG-3′. The comparative Ct (ΔΔCt) method was used to analyze fold changes in expression using GAPDH as an internal control.

### Western blot analysis

Cells were harvested after washing twice with PBS, and cells were lysed with RIPA lysis buffer (50 mM Tris,pH 7.4, 150 mM NaCl, 1% NP-40, 0.5% sodium deoxycholate, and 0.1% SDS) in the presence of a protease inhibitor cocktail (Kangcheng, Shanghai, China). Protein concentration was determined using a BCA protein assay kit (Beyotime Institute of Biotechnology, China). Samples with equal amount of protein was boiled at 95 °C for 10 min after adding 2 x SDS-PAGE sample loading buffer (62.5 mM Tris-Cl pH 6.8, 2% SDS, 10% glycerol, β-mercaptoethanol, and 0.002% bromophenol blue). For Western blotting, 60 μg of total protein were separated by SDS-PAGE, and transferred to PVDF membranes (Amersham Biosciences, Pisctaway NJ). Blots were then blocked in 5% nonfat milk at room temperature for 1 h and incubation overnight at 4 °C with anti-SHH (1:1000, Rabbit, Cell Signaling Technology), anti-GLI1 (1:1000, Rabbit, Cell Signaling Technology), anti-Cyclin D1 (1:1000, Rabbit, Cell Signaling Technology), anti-P27 Kip1 (1:1000, Rabbit, Cell Signaling Technology) anti-CDK6 (1:1000, Mouse, Cell Signaling Technology) anti-P21 Waf1/Cip (1:1000, Rabbit, Cell Signaling Technology) anti-CDK4 (1:1000, Rabbit, Abcam), anti-Cleaved PARP (1:1000, Rabbit, Cell Signaling Technology) and anti-GAPDH (1:3000, Mouse, Santa) antibodies. The horseradish peroxidase-conjugated secondary antibodies (1:5000; KangChen Bio-tech, China) were used to incubate the membranes. GAPDH was used as a protein loading control, protein signals were detected by chemiluminescence.

### Dual luciferase assay

MKN45 and AGS cells grown in 24-well plates were co-transfected with 10 ng of pRL-TK and 200 ng Gli1, Smo promoter-driven luciferase reporter vector (Gli1-pGL3, Smo-pGL3) or the luciferase reporter plasmid pGL3-Basic vector with FuGene transfection reagent (Promega). Itraconazole or DMSO was added 6 h after transfection. The cells were harvested 48 h after transfection and luciferase assays were performed using the dual luciferase assay kit (Promega) according to the manufacturer’s instructions. Luciferase activities were normalized to those of Renilla luciferase and the activity of pGL3-basic vector was set at 1. Experiments were carried out in triplicate.

### Tissue microarrays and immunohistochemistry

Tissue arrays (HStm-Ade180Sur-06) were obtained from the Shanghai Biochip Company (Shanghai Outdo Biotech Co. Ltd, China). Ninety pairs of gastric cancer tissues and adjacent normal counterparts were included. Arrays were incubated in 0.3% hydrogen peroxide for 15 min at room temperature to suppress endogenous peroxidases,and antigen retrieval was performed in a pressure cooker with Tris–EDTA buffer. Tissue sections were incubated with anti-Shh rabbit monoclonal antibody (1:400, Cell Signaling Technology) and anti-Gli1 rabbit monoclonal antibody (1:500, Cell Signaling Technology) at 4 °C overnight. Secondary antibody was applied using Polymer-HRP Kit. After the diaminobenzidine (DAB) reaction was developed, the slides were then counterstained with hematoxylin.

The immunostaining results of every tissue point was evaluated by combining the percentage of positive cells (quantity score) with the intensity of staining (staining intensity score). For the quantity score, no staining is scored as 0, 1–10% of cells stained scored as 1, 11–50% as 2, 51–80% as 3 and 81–100% as 4. Staining intensity is scored as score 0 for no staining; score 1 for weak staining; score 2 for moderate staining and score 3 for strong staining. The final staining score is given by multiplying the quantity and staining intensity scores. Therefore, the scores could range from 0 to 12. An IHS score less than 6 was considered as low expression, and a scole of 6 or greater was categorized as high expression.

### Tumor xenograft model

To generate subcutaneous tumor, 5 × 10^6^ AGS or MKN45 cells were suspended in 100 mL of PBS and injected subcutaneously into the right flank of male nude mice (BALB/c, 4 to 6 weeks of age). When tumors reached a size measuring 40 to 70 mm^3^, calculated as V = L × W^2^/2(V = Tumor volume, L = Length, W = Width), mice were treated with hydroxypropyl-cyclodextrin (vehicle control), itraconazole (75 mg/kg twice daily by oral administration), 5-FU (20 mg/kg every 2 days by intraperitoneal injection), or a combination of itraconazole and 5-FU. All treatments were administered in 10 mL/kg volumes with 12-h separation between bi-daily treatments. Tumors were measured three times per week for 2 to 3 weeks. Then mice were sacrificed and tumor tissues were isolated and frozen in liquid nitrogen or fixed in formalin immediately.

### Statistical analysis

Student’s *t*-test was used for statistical analysis of the differences between the mean values of two groups. The Chi-square test or Fisher’s exact test was used to determine the strength of correlations among protein expressions and clinicopathological factors. Overall survival was determined using the Kaplan-Meier method. A two-sided *p*-value of <0.05 was considered statistically significant in all tests.

## Results

### Itraconazole inhibits proliferation of gastric cancer cells and enhances the chemotherapeutic response of 5-FU in vitro and in vivo

**Fig. 1:**
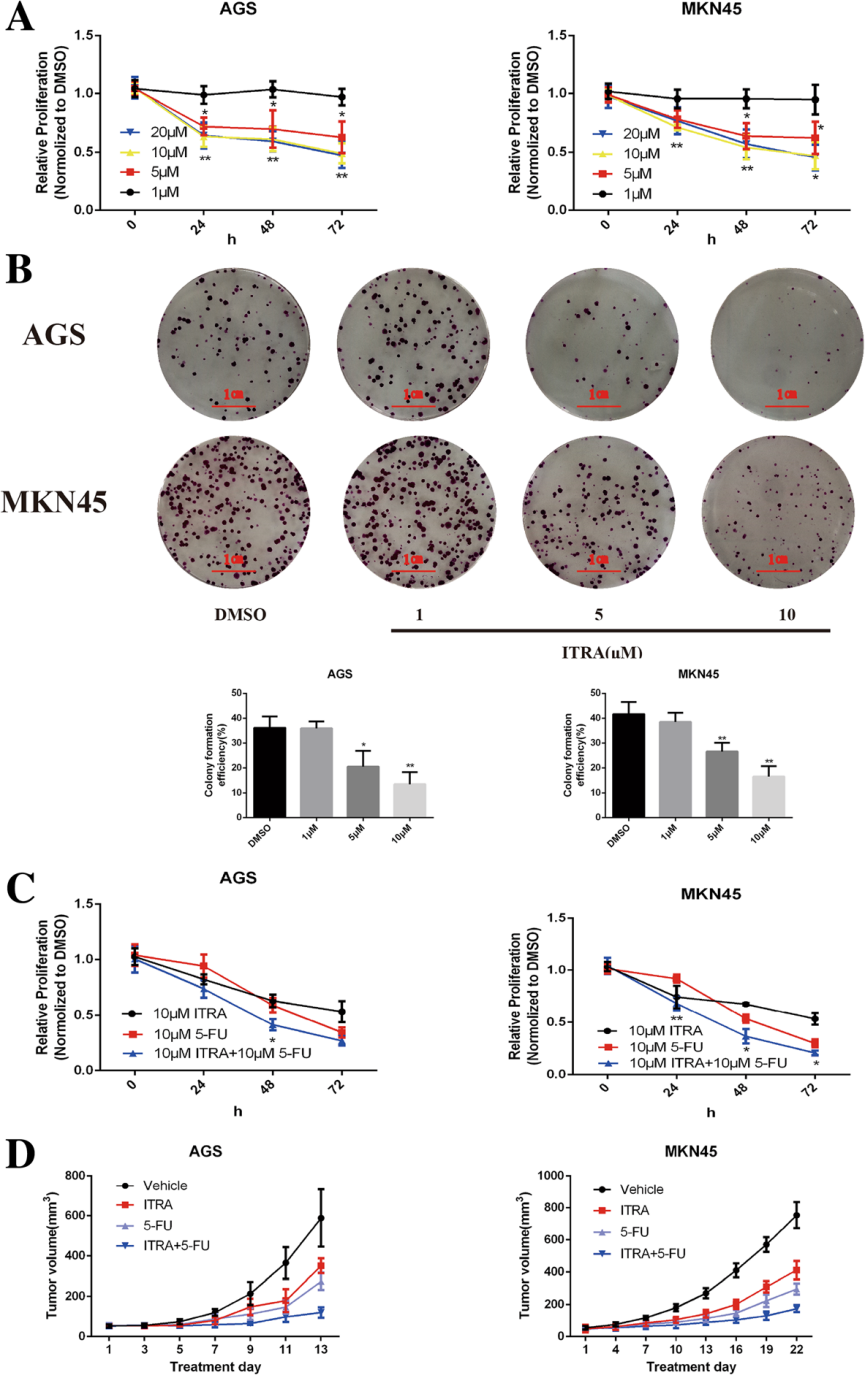
Itraconazole inhibits the growth of gastric cancer cells in vitro and in vivo.**a** Decrease in viability of gastric cancer cells (AGS and MKN45) following treatment with increasing concentrations of itraconazole. Cell viability was measured by the CCK-8 assay. Data represent the mean of three experiments (mean ± SD). *p < 0.05; **P <0.01 vs DMSO-treated cells. ITRA, itraconazole. **b** Repression of colony formation of gastric cancer cells following treatment with different concentrations of itraconazole. AGS and MKN45 cells, treated with itraconazole, were grown in a 6-well plate and colonies were scored after 14 days. Cell clusters containing more than 50 cells under a microscope were considered as colonies. Histograms show the formation rate of colonies. Data represent the mean of three experiments (mean ± SD). *P <0.05 and **P <0.01, vs DMSO treated cells. **c** Itraconazole enhances the inhibitory effect of 5-FU on gastric cancer cells. AGS and MKN45 cells were treated with 10 μM itraconazole, 10 μM 5-FU or both. Data represent the average of three experiments (mean ± SD). *P <0.05 and **P <0.01 vs DMSO-treated cells. **d** Itraconazole inhibits growth of gastric cancer xenografts. Nude mice with AGS subcutaneous tumor xenografts were treated with vehicle (*n* = 8), itraconazole 75 mg/kg twice daily by oral administration (ITRA; *n* = 8), 5-FU 20 mg/kg every 2 days by intraperitoneal injection (*n* = 8), or a combination of itraconazole and 5-FU (ITRA + 5-FU; *n* = 6). Nude mice with MKN45 subcutaneous tumor xenografts were treated with vehicle (*n* = 10), itraconazole 75 mg/kg twice daily by oral administration, 5-FU 20 mg/kg every 2 days by intraperitoneal injection (*n* = 8), or a combination of itraconazole and 5-FU (ITRA + 5-FU; *n* = 8). Mean ± SD tumor volumes are reported for each treatment group

In order to verify the potential anti-cancer properties of itraconazole, we firstly tested whether it could inhibit the proliferation of gastric cancer cells. To this end, MKN45 and AGS cells were treated with increasing concentrations of itraconazole and cell proliferation was determined with CCK-8 assay. As shown in Fig. 1a, itraconazole could inhibit cell proliferation in a dose-dependent manner in both cell lines. Furthermore, colony formation assay showed that itraconazole could dramatically inhibit the colony formation in a concentration dependent manner, reflected by fewer and smaller colonies in the drug-treated group (Fig. 1b). More interestingly, when cells were treated with 5-FU and itraconazole concomitantly, itraconazole could greatly increase the cytotoxicity of 5-FU to gastric cancer cells (Fig. 1c). The antitumor properties of itraconazole in gastric cancer were further evaluated using tumor xenograft model. As shown in Fig. 1d, Itraconazole inhibited growth of AGS and MKN45 cells xenografts as a single agent and in combination with 5-FU therapy. Taken together, these data suggested that itraconazole acts as a proliferation suppressor in gastric cancer cells.

### Itraconazole regulates the G_1_-S transition and induces apoptosis of gastric cancer cells

**Fig. 2:**
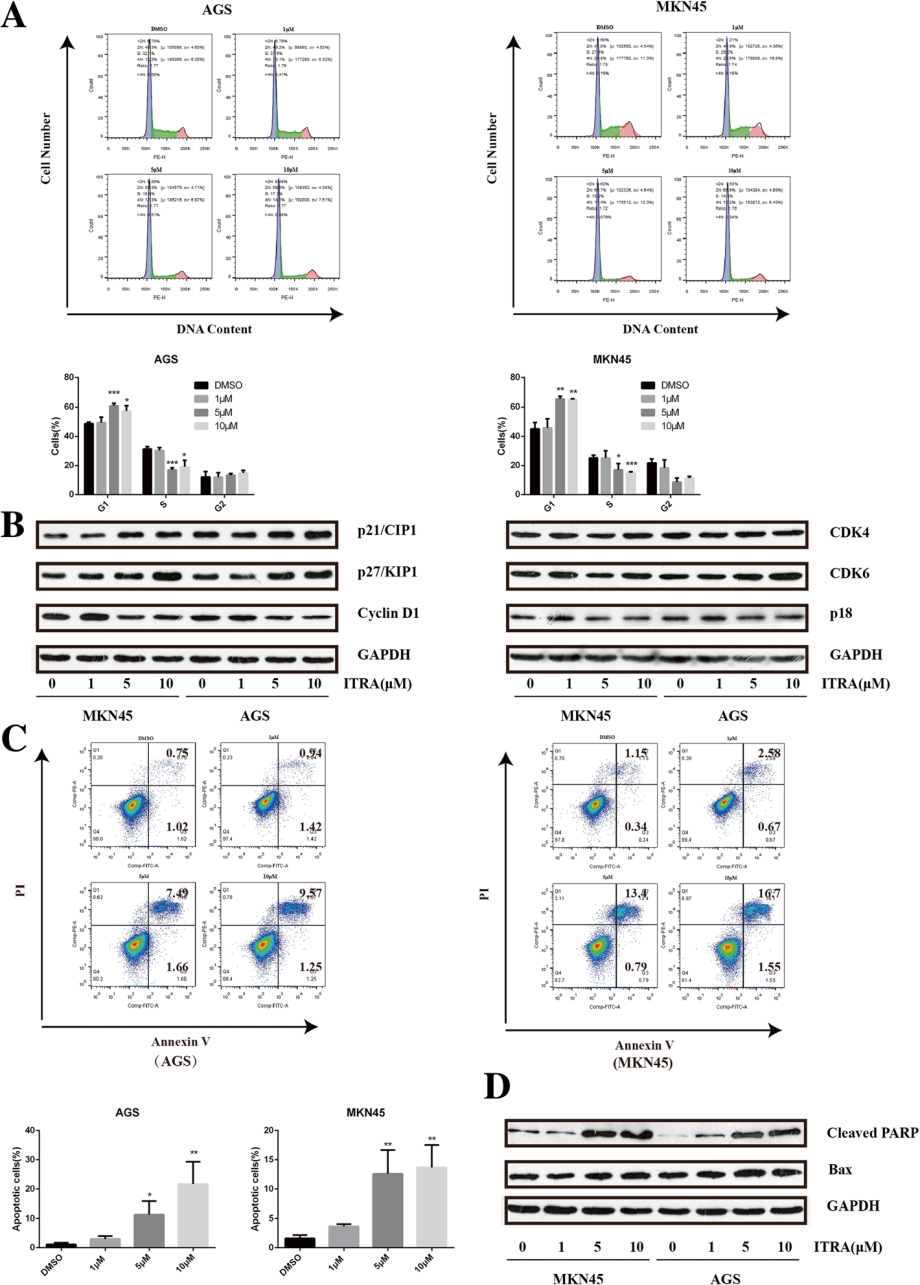
Effects of itraconazole on cell cycle distribution and apoptosis of gastric cancer cells. **a** Cell cycle distributions were detected by flow cytometry analysis in AGS and MKN45 cells after treatment with different concentrations of itraconazole or DMSO for 72 h. Data represent the mean of three experiments (mean ± SD). *p < 0.05; **P <0.01, compared to DMSO treated cells. **b** The expression of cell cycle-related proteins is examined by immunoblot assay. AGS and MKN45 cells are harvested after itraconazole treatment for 48 h. GAPDH is used as a loading control. **c** Cell apoptosis is determined by flow cytometry analysis in AGS and MKN45 cells after treatment with different concentrations of itraconazole or DMSO for 72 h. Data represent the average of three experiments (mean ± SD). *P <0.05 and **P <0.01 vs DMSO treated cells. **d** The Bax expression at protein level is examined by immunoblot assay. GAPDH is used as a loading control

Cell proliferation inhibition often accompanies changes in cell cycle progression [23], therefore, we next evaluated cell cycle distributions in MKN45 and AGS cells. After treatment with itraconazole for 72 h, the cell cycle distribution changed significantly, with a significant increase in cell population G_1_ phase and a decrease in cell population in S phase (Figs. 2a). In MKN45 cells, for example, the G_0_/G_1_–phase fraction increased from 45.1% (DMSO treated) to 45.8, 65.4, and 65.1% when cells were treated with 1, 5, and 10 μM itraconazole, respectively. Moreover, Western blot showed that the expression of cell cycle-related proteins, such as p21Waf1/Cip1(p21), p27KIP1(p27) and Cyclin D1, were changed dramatically after itraconazole treatment for 48 h. As shown in Fig. 2b, the expression level of p21 and p27 were markedly increased while cyclin D1 protein was reduced in itraconazole-treated groups compared to control group. The two main cyclin-dependent kinase inhibitors, p21 and p27, are ceased during the entry to S-phase [24]. The activation of Cyclin D1 mainly regulates the G1-S phase transition [25].

Furthermore, we also investigated whether itraconazole could induce apoptosis, an important mechanism of antitumor drugs, in gastric cancer cells. Apoptosis cells were analyzed with Annexin V-propidium iodide (PI) staining and flow cytometry. As shown in Fig. 2c, itraconazole could significantly induce apoptosis of MKN45 and AGS cells, with an 8.56-fold and 15.5-fold increase in apoptosis cells in MKN45 and AGS cells after treatment with 10 μM itraconazole for 72 h. Consistently, the expression of Bax, the main apoptosis promoting protein in the Bcl-2 protein family and cleaved PARP, a sensitive apoptotic marker, were increased after itraconazole treatment for 72 h (Fig. 2d).

These findings suggest that itraconazole not only inhibits cell proliferation through regulation of the G1-S transition but also induces apoptosis in gastric cancer cells.

### Itraconazole regulates Hh signaling by inhibition of Gli1 transcription

**Fig. 3:**
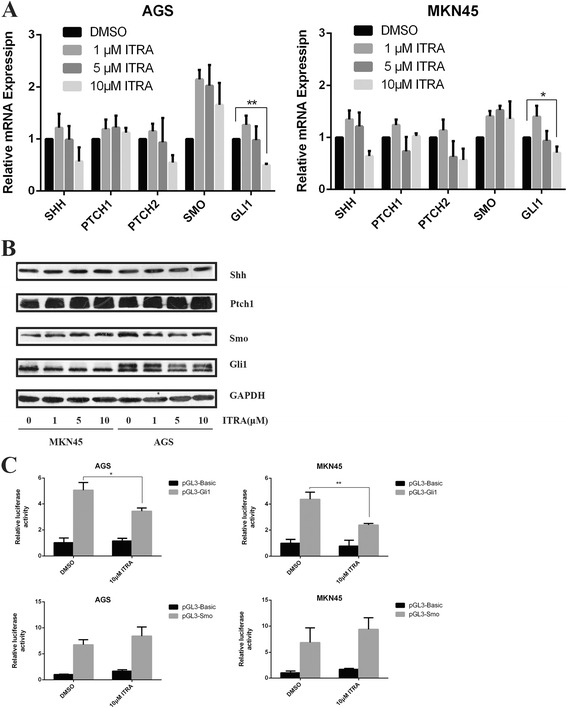
Itraconazole inhibits the expression of Gli1 in gastric cancer cells. **a** Expressions of Shh, Ptch, Smo, and Gli1 in MKN45 and AGS cells treated with increasing concentrations of itraconazole for 48 h. Data represent the mean ± SD of 3 determinations and GAPDH is used as the internal control. *P < 0.05; **p < 0.01. **b** MKN45 and AGS cells are treated with DMSO or indicated concentrations of itraconazole for 48 h, and expressions of major molecules of Hh signaling are determined by immunoblot. A representative blot from three independent experiments is shown. **c** MKN45 and AGS cells are transiently transfected with Gli1-pGL3, Smo -pGL3 or pGL3-Basic vector and pRL-TK, followed by treatment with 10 μM itraconazole or DMSO. After 48 h, Gli1-pGL3, Smo -pGL3 or pGL3-Basic vector reporter activities were measured and normalized to Renilla luciferase activity. Data represent the mean of three experiments (mean ± SD). *p < 0.05 and **P <0.01 vs DMSO treated cells

Many studies indicated that the anti-cancer properties of itraconazole are closely related to Hh signal pathway [16, 22, 26, 27]. Hence, we investigated the effect of itraconazole on the expression of Hh-related molecules, including Shh, Ptched1, Ptched2, Smo and Gli1, in gastric cancer cells. After treatment with itraconazole for 48 h, the changes of the components of Hh pathway at mRNA and protein levels were determined by real-time RT-PCR and Western blotting. The results revealed that the mRNA level of Gli1, indicating a constitutive activation of the Hh pathway [28], was reduced with the treatment of itraconazole. However, other components, especially Smo, which had been thought to be the target of itraconazole [22, 27], showed no significant changes (Fig. 3a). Consistent with mRNA expression, we also observed that the protein level of Gli1 was decreased and Smo was unchanged in itraconazole-treated gastric cancer cells (Fig. 3b). For further validation, a dual luciferase assay was performed 48 h after treatment with the indicated reagents. We found that 10 μM itraconazole decreased Gli1-pGL3 luciferase activity compared to DMSO treated cells (Fig. 3c). These data suggest that itraconazole might directly or indirectly act on Gli1 instead of Smo to inhibit Hh signal pathway in gastric cancer cells.

### Hh signal pathway is activated in human gastric cancer tissues

**Fig. 4:**
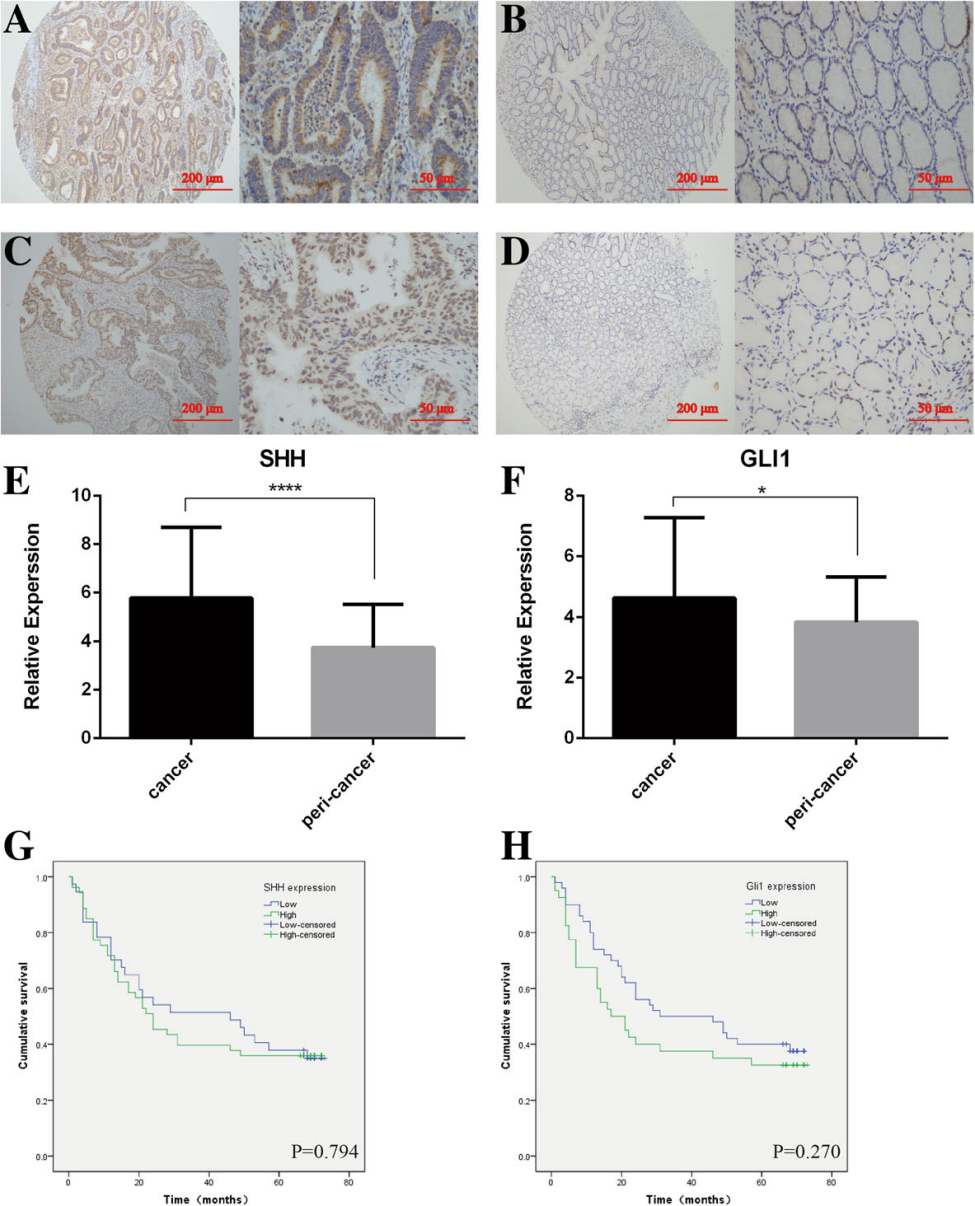
Immunostaining analysis of the Hh signaling molecules (SHH and GLI1) in human gastric cancer tissues. SHH expression is mainly expressed in the cytoplasm and GLI1 protein is strongly localized to the nucleus and cytoplasm in gastric cancer cells. The micrographs showed representative immunohistochemical staining of SHH (**a**) and GLI1 (**c**) in the gastric cancer tissues. The expression of SHH and GLI1 in corresponding adjacent normal gastric tissues is shown in (**b**) and (**d**) respectively (magnification: *left panel* × 100, right panel × 400). Quantitative analysis of SHH (**e**) and GLI1 (**f**) in tumor tissues and adjacent normal gastric tissues. *p < 0.05 and ***P <0.001 for SHH and GLI1 vs their respective controls, respectively. The differences in overall survival time between high and low expression of SHH (**g**) and GLI1 (**h**) in gastric cancer tissue are determined using the Kaplan-Meier method

In order to examine whether Hh signaling pathway was activated in gastric cancer, we determined the expression of SHH and GLI1 in 90 pairs of gastric cancer tissue and adjacent normal tissue samples by immunohistochemistry. SHH was mainly expressed in the cytoplasm (Fig. 4a) and GLI1 protein was predominantly localized to the nucleus and cytoplasm in gastric cancer cells (Fig. 4c). In gastric cancer tissue samples, we detected higher levels of SHH expression than in the normal tissues (Fig. 4e). When Hh signaling was activated, the expression of Gli1 was up-regulated and it was translocated into the nucleus [28, 29]. The percentage of cells with nuclear and cytoplasm staining of GLI1 was higher in cancer tissues than that in the adjacent normal tissues (Fig. 4f). These data suggest that Hh signal pathway is activated in human gastric cancer tissues.

To determine the correlations between the expressions of SHH and GLI1 and clinicopathologic factors, patients were divided into high- and low-expressing groups, according to the median expression of each target. We found that expression of GLI1 was significantly correlated with age (*p* < 0.05), however, there was no significant association between SHH protein overexpression and these clinicopathologic variables (gender, age, tumor size, distant metastasis, local invasion and TNM stage) (*p* > 0.05). (Table 1) Though there were no significant differences, we found that overall survival time was shorter in patients with high expression levels of SHH and GLI1 than that in patients with low expression levels (Fig. 4g and h). To predict patient prognosis, univariate and multivariate Cox regression analyses were performed. The significant prognostic factors in univariate analysis included tumor size, lymphatic invasion, vessel invasion, distal metastasis, and depth of invasion (*p* < 0.05). Multivariate analysis revealed that lymphatic invasion and distal metastasis were independently associated significantly with the decreased survival.

**Table 1:** Correlations between expressions of SHH and GLI1 protein and clinicopathological factors

	SHH expression				GLI1 expression			
Clinical parameters	Low (*n* = 37)	High (*n* = 53)	χ^2^	*p*	Low (*n* = 50)	High (*n* = 40)	χ^2^	*p*
Gender								
Male	20	33	0.607	0.436	24	29	5.510	0.019*
Female	17	20			26	11		
Age								
< 60 years	13	22	0.373	0.542	15	20	3.740	0.053
≥ 60 years	24	31			35	20		
Tumor size								
< 5.0 cm	14	21	0.029	0.864	21	14	0.458	0.498
≥ 5.0 cm	23	32			29	26		
Tumor Location								
Fundus	5	6	0.241	0.886	6	5	0.477	0.788
Body	15	20			21	14		
Antrum	17	27			23	21		
Invasion depth								
T1,T2	4	10	1.077	0.383	11	3	3.557	0.080
T3,T4	33	43			39	37		
LNM								
Negative	11	12	0.575	0.448	13	10	0.012	0.914
Positive	26	41			37	30		
Metastasis								
Absent	35	51	0.137	1.000	48	38	0.052	1.000
Positive	2	2			2	2		
Vessel invasion								
Negative	28	33	1.795	0.180	36	25	0.918	0.338
Positive	9	20			14	15		
TNM stage								
I, II	14	26	1.111	0.292	21	19	0.272	0.602
III, IV	23	27			29	21		

*TNM* Tumor, node, metastasis, *LNM* Lymph node metastasis

^*^Significant difference

## Discussion

Our results suggest that Hh signal pathway is activated in human gastric cancer and itraconazole can inhibit gastric cancer cells growth by inhibiting the expression of Gli1. The expression of Gli1, a marker of Hh signaling activation, was higher in gastric cancer tissue than that in the adjacent normal tissue in our study. Several studies indicated Helicobacter pylori infection activates the Hh signaling pathway through the up-regulation of Shh in gastric cancer [30–32], which is consistent with our results. The overall survival time was shorter in patients with high expression levels of Gli1 than that in patients with low expression levels though the difference was of no statistical significant, which may be attributed to the limited sample size. The results of this study indicated that 10 μM itraconazole could inhibit the proliferation of gastric cancer cells by about 50% after 48 h. When the cells were treated with 10 μM 5-FU in combination with 10 μM itraconazole, the inhibitory effect of 5-FU could be significantly enhanced by itraconazole, reflected by an further 20% decrease in the proliferation of gastric cancer cells compared with that treated with 10 μM 5-FU alone. In xenograft models of AGS or MKN45 cells, itraconazole could not only inhibit the growth of tumors as a monotherapy but also enhance the therapeutic effect of 5-FU. We also observed the populations of cells were significantly increased in G1 phase and decreased (about 10%) in S phase after treatment with 10 μM itraconazole for 72 h. In addition, it was shown in this study that 10 μM itraconazole could increase 15% of apoptosis rate of gastric cancer cells compared with the control group. Taken together, itraconazole could greatly inhibit the growth of gastric cancer cells and it may predict that itraconazole can be a good therapeutic drug in gastric cancer.

**Fig. 5:**
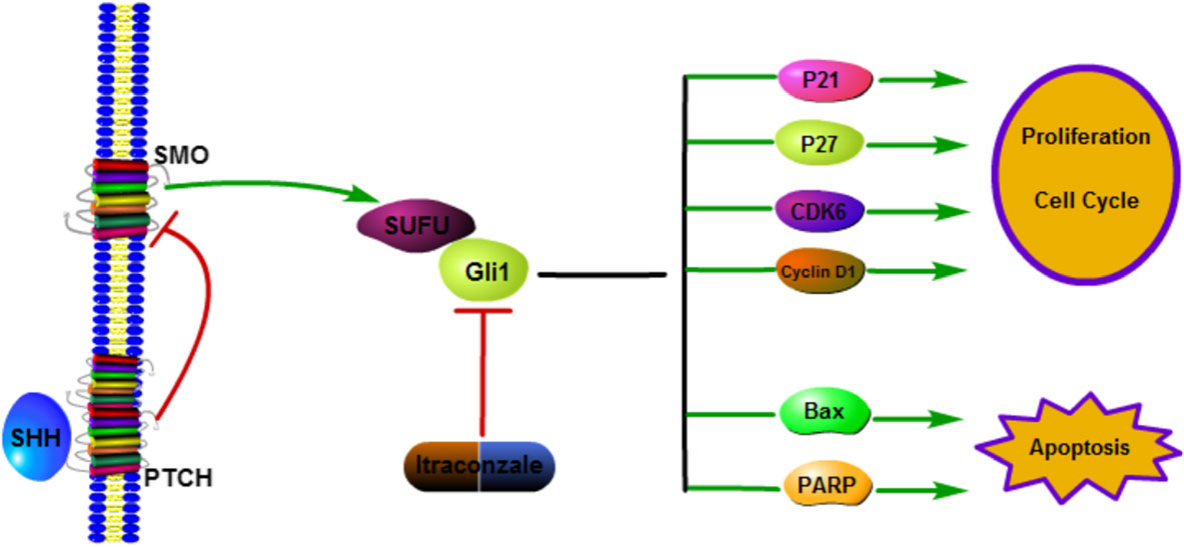
Diagram of the putative effects of itraconazole on gastric cancer cells. Itraconazole down-regulates the expression of Gli1 by yet unknown mechanisms and subsequently regulates the expression of several downstream target genes, including p21^waf1/cip1^, and p27^kip1^, Bax and PARP, thereby regulating many cellular processes, such as proliferation, survival, cell cycle and apoptosis

Since it has been found that itraconazole could inhibit Hh signaling [11, 22, 33], many in vitro and in vivo studies have been conducted to evaluate the putative antitumor activities of itraconazole [13, 14, 16, 17, 19, 34–36]. Indeed, it has been shown that itraconazole is associated with a trend of improved disease control in patients with various cancers. However, our results showed that itraconazole had no inhibiting effect on SMO expression at mRNA and protein levels, which was inconsistent with previous reports. Kim et al. thought itraconazole may act on Smo at a distinct site from that of cyclopamine [22], which is able to bind Smo directly [37–39]. But they did not demonstrate the direct binding of itraconazole to Smo. Therefore, it is possible that itraconazole may act on Smo through other indirect mechanisms. Moreover, they confirmed that itraconazole may function as an Hh pathway antagonist by decreasing GLI expression in the tumor [14]. In our study, we showed that itraconazole could inhibit gastric cancer cells growth by regulating the expression of Gli1, which is in accordance with a previous study. The authors proved that the growth inhibitory effect induced by activation of Hh signaling is mainly through the Gli1 protein, as shown in Fig. 5, and Gli1 plays a pivotal role in cell proliferation through regulation of the G_1_-S transition and that Gli1 is implicated in the Shh-dependent autocrine loop that accelerates the proliferation of gastric carcinoma cells [40]. Itraconazole has no effects on other parts of Hh signaling, indicating that itraconazole may impact Gli1activation by non-canonical Hh signaling pathways. Emerged evidence shows that there are non-canonical pathways capable of activating Gli1, e.g., TGFβ and RAS, and implicating activation of downstream Hh target genes by other ligands [41, 42].

Itraconazole, once established its antitumor activity, will play an important role in gastric cancer treatment for its relative low price and well documented safety and side effects. Previous studies suggested that itraconazole may inhibit tumors by regulating Hh signaling. Therefore, it should be of significance to evaluate the combined effect of itraconazole with other targeted therapies or cytotoxic agents in patients with gastric tumors. In this study, it was shown that itraconazole was effective in the inhibiting cell proliferation and could improve the chemotherapeutic effect of 5-FU in vitro and in vivo; however, the exact antitumor effects of itraconazole still need to be verified in animal studies and if possible in clinical studies.

## Conclusion

Hh signaling is activated in gastric cancer tissues; inhibition of Gli1 expression might be at least partially responsible for the proliferation inhibitory effect of itraconazole in gastric cancer cells, which needs to be verified in the future. In addition, the underlying mechanisms of Gli1 inhibition by itraconazole are largely unknown, which also need to be explored in the further studies in order to better understand the molecular basis of itraconazole action in cancer cells. Our findings support the scenario that the traditional antifungal drug itraconazole might be of the potential as an antitumor agent in gastric cancers.

## Abbreviations

GC: Gastric cancer
Gli1: Glioma-associated zinc finger transcription factor 1
Hh: Hedgehog
